# OrthoVenn3: an integrated platform for exploring and visualizing orthologous data across genomes

**DOI:** 10.1093/nar/gkad313

**Published:** 2023-04-28

**Authors:** Jiahe Sun, Fang Lu, Yongjiang Luo, Lingzi Bie, Ling Xu, Yi Wang

**Affiliations:** Integrative Science Center of Germplasm Creation in Western China (CHONGQING) Science City, Biological Science Research Center, Southwest University, Chongqing, China; Integrative Science Center of Germplasm Creation in Western China (CHONGQING) Science City, Biological Science Research Center, Southwest University, Chongqing, China; Integrative Science Center of Germplasm Creation in Western China (CHONGQING) Science City, Biological Science Research Center, Southwest University, Chongqing, China; Integrative Science Center of Germplasm Creation in Western China (CHONGQING) Science City, Biological Science Research Center, Southwest University, Chongqing, China; State Key Laboratory of Plant Environmental Resilience, College of Biological Sciences, China Agricultural University, Beijing, China; Integrative Science Center of Germplasm Creation in Western China (CHONGQING) Science City, Biological Science Research Center, Southwest University, Chongqing, China

## Abstract

Advancements in comparative genomics research have led to a growing interest in studying species evolution and genetic diversity. To facilitate this research, OrthoVenn3 has been developed as a powerful, web-based tool that enables users to efficiently identify and annotate orthologous clusters and infer phylogenetic relationships across a range of species. The latest upgrade of OrthoVenn includes several important new features, including enhanced orthologous cluster identification accuracy, improved visualization capabilities for numerous sets of data, and wrapped phylogenetic analysis. Furthermore, OrthoVenn3 now provides gene family contraction and expansion analysis to support researchers better understanding the evolutionary history of gene families, as well as collinearity analysis to detect conserved and variable genomic structures. With its intuitive user interface and robust functionality, OrthoVenn3 is a valuable resource for comparative genomics research. The tool is freely accessible at https://orthovenn3.bioinfotoolkits.net.

## Graphical Abstract

**Graphical Abstract:**
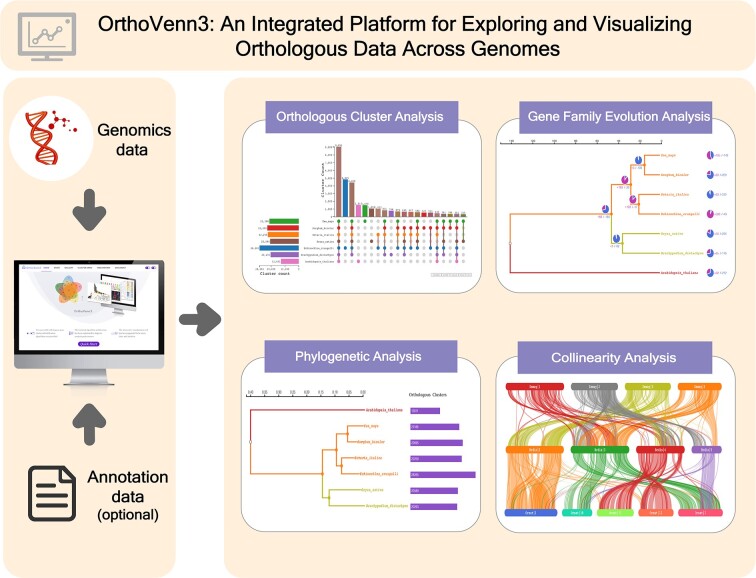
OrthoVenn3 accepts genomics data and optional gene annotation information for orthologous cluster analysis, phylogenetic analysis, gene family evolution analysis and collinearity analysis.

## INTRODUCTION

Comparative genomics studies have emerged as a critical area of research in the life sciences due to the explosive growth of genome sequencing (1,2). It can be performed at different aspects of the genome and obtain multiple viewpoints about the organisms (3–5). To facilitate this type of analysis, OrthoVenn, an online whole-genome comparative analysis tool, was developed. First released in 2015 and updated in 2019, both versions of OrthoVenn were published in the *Nucleic Acids Research* web server issue (6,7). OrthoVenn automates the identification and annotation of orthologous clusters, offering rich data visualization through occurrence tables, Venn diagrams, network diagrams, and so on, drawing broad attention and widespread usage.

This paper introduces a new version of OrthoVenn with multiple updates that improve its functionality for comparative genomics research. First, we increased the data capacity to include more species and added gene annotation information. Second, we integrated OrthoFinder2 (8), a widely used method for identifying orthologous clusters, to enhance the accuracy of OrthoVenn3. Third, we incorporated UpSet, a tool for visualizing set intersections in a matrix layout (9), making it easier to identify unique and shared clusters among numerous species. UpSet is well suited for the quantitative analysis of species with more than six sets. In addition to these features, OrthoVenn3 wraps new tools for comprehensive comparative genomics analysis, including (i) phylogenetic analysis, allowing for the inference of evolutionary relationships among species based on their orthologous clusters, (ii) gene family contraction and expansion analysis, which provides insight into the gain or loss of gene families among different species and (iii) collinearity analysis, which helps to identify regions of genomic rearrangement and evolution.

In summary, OrthoVenn3 is an effective and user-friendly online tool for comparative genomics research, providing researchers with intuitive data visualization and diverse analysis capabilities. The tool requires protein sequences in fasta format as input, with optional gene annotation information in bed format. OrthoVenn3 offers multiple outputs, including the UpSet table, occurrence table, phylogenetic tree, and collinearity graph, which provides users with various perspectives on their data. As an illustration, we conducted interspecies comparative analysis on seven plant species to showcase the utility of OrthoVenn3. The results of this analysis are discussed in the ‘CASE STUDY’ section, highlighting the tool's ability to identify orthologous clusters, visualize their distribution across different species and infer evolutionary relationships. Overall, OrthoVenn3 is a comprehensive and versatile tool for comparative genomics research, potentially advancing our understanding of the evolutionary relationships and genetic diversity among diverse species.

## DATA HUB UPDATES

OrthoVenn3 has expanded its built-in database, which is sourced from the Ensembl database (2022 version) (10), resulting in an increase in the number of species from 540 to 733. This expansion provides access to a total of 11 960 346 protein sequences. Additionally, OrthoVenn3 also added gene annotation information for protein sequence as a new feature. To provide easy access to this information, protein sequence and gene annotation data for each species are stored separately in six built-in databases for vertebrates (164 species), metazoa (107 species), protists (90 species), fungi (139 species), plants (92 species) and bacteria (141 species). To simplify the search for species of interest, OrthoVenn3 has introduced a search box for species, enabling users to search for and add species by name. Furthermore, to ensure the accuracy of species information, we intend to regularly update species information, aligning our update frequency with that of the Ensembl database. Overall, these developments offer users improved functionality, greater accessibility, and more comprehensive coverage, thereby increasing the utility of OrthoVenn3 as a tool for comparative genomics research.

## NEW FEATURES

### Orthologous cluster identification algorithm and visualization

**Figure 1.**
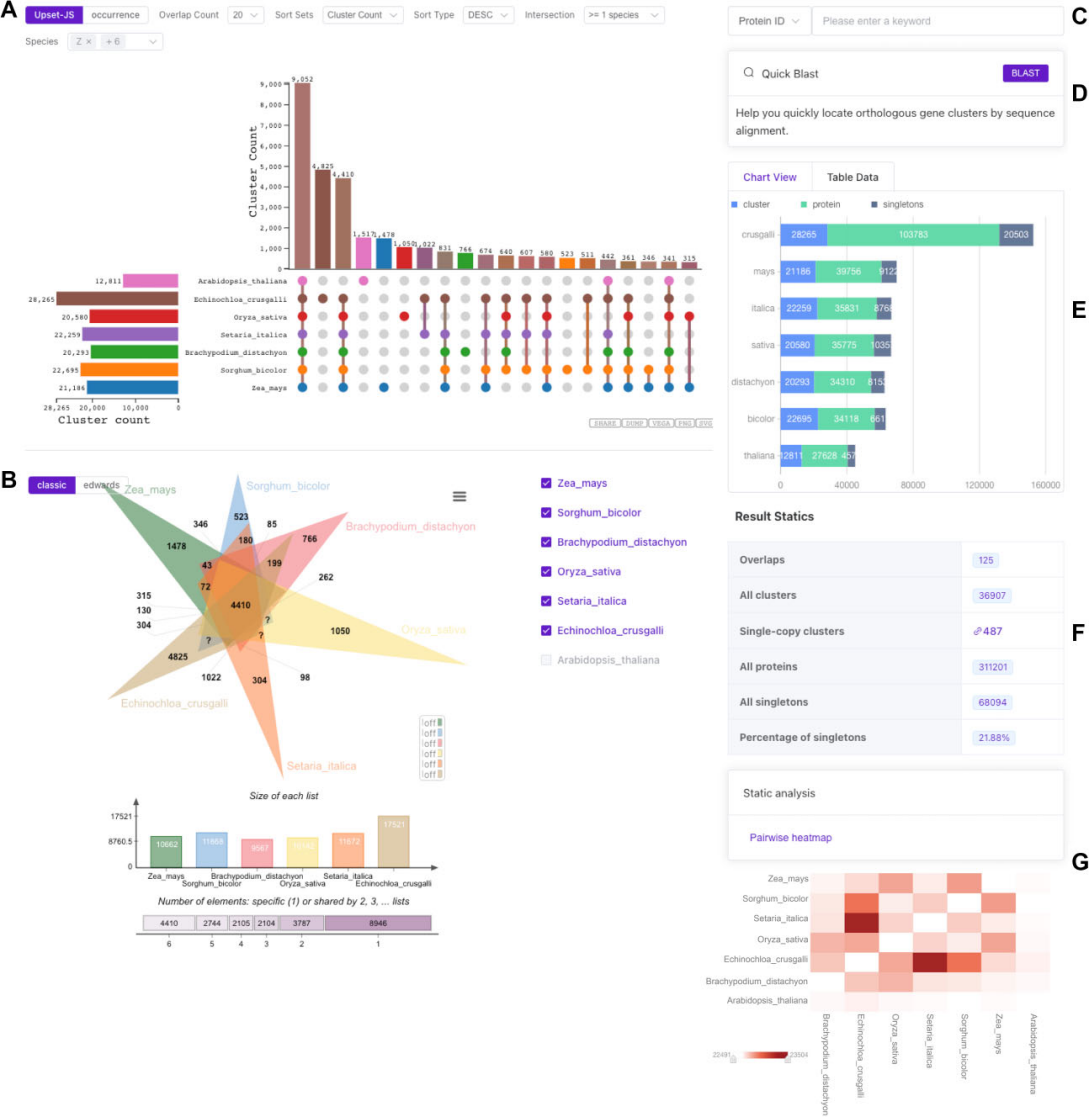
Orthologous cluster analysis of seven plant species: *Echinochloa crusgalli*, *Setaria italica*, *Zea mays*, *Sorghum bicolor*, *Brachypodium distachyon*, *Oryza sativa* and *Arabidopsis thaliana*. (**A**) An UpSet table displays unique and shared orthologous clusters among the species. The left horizontal bar chart shows the number of orthologous clusters per species, while the right vertical bar chart shows the number of orthologous clusters shared among the species. The lines represent intersecting sets. (**B**) Classic Venn and Edwards diagrams show selected species. (**C**) Retrieve orthologous clusters quickly using either a Cluster ID or a protein ID. (**D**) Upload nucleotide or protein sequences for sequence alignment with the output cluster. (**E**) The bar chart shows the number of protein sequences, orthologous clusters and singletons for each species. (**F**) The table shows the total number of sets, clusters, single-copy gene clusters, singletons and the percentage of singletons across all species. (**G**) The heatmap shows the number of overlapping clusters between each pair of species.

Identifying orthologous clusters is critical for comparative genomic studies (11,12), as it enables the comparison of evolutionary relationships between genes across different species (13). To improve the accuracy and efficiency of this process, OrthoVenn3 has incorporated the OrthoFinder algorithm, which is the most balanced orthologous gene cluster identification algorithm according to the latest benchmark test results of Quest for Orthologs (14). However, the classic approach - Venn Diagram cannot effectively visualize more than six intersecting sets. Hence, OrthoVenn3 has adopted UpSet tables (9) for data visualization, which allows for viewing pairwise intersections between sets with >30 sets or more. The data visualization methods employed by OrthoVenn3 are particularly effective for analyzing numerous species, providing users with a variety of options for exploring the results of orthologous gene cluster identification. The UpSet table presents the number of orthologous clusters in each species, as well as the number of shared orthologous gene clusters among species, in a clear and intuitive format (Figure 1A). By clicking on the nodes of the UpSet table, users can access Gene Ontology (GO) (32) term annotations for individual orthologous clusters, allowing for more detailed analysis of the results.

OrthoVenn3 also provides users with the ability to select target species for visualization, as well as two visualization modes: UpSet and occurrence tables. The use of bar charts (Figure 1E) to display the number of genes, orthologous groups, and singletons in each species helps users to better interpret the analysis results. Additionally, the identification of single-copy gene clusters (Figure 1F) is supported, and users can access GO term annotations for these clusters by clicking on the relevant number. OrthoVenn3 further offers a Cluster ID and Protein ID search function (Figure 1C) that allows users to retrieve annotation information from the results of orthologous clusters. The Blast module allows for the upload of protein or nucleotide sequences for comparison with the output results (Figure 1D). Finally, users can select up to 6 species to draw classic Venn diagrams or Edwards diagrams (Figure 1B), and the heatmap module (Figure 1G) supports viewing the shared orthologous gene clusters between species.

### Phylogenetic analysis function

Phylogenetic analysis is widely used in species classification, phylogenetic reconstruction, and inference of evolutionary history (15,16). In response to user's feedback, we have added a phylogenetic analysis function in the latest version of OrthoVenn3. We used FastTree (18) to construct phylogenetic trees based on conserved single-copy gene sequences. The single-copy gene clusters contain only one gene from each species are considered independent evolutionary units among species (17). FastTree employs the maximum likelihood method (19) to analyze a large number of sequences at a faster speed, ensuring accuracy while significantly reducing analysis time. This makes it possible to construct phylogenetic trees with large datasets of multiple species and helps readers more accurately understand biodiversity's origin and evolutionary history.

**Figure 2.**
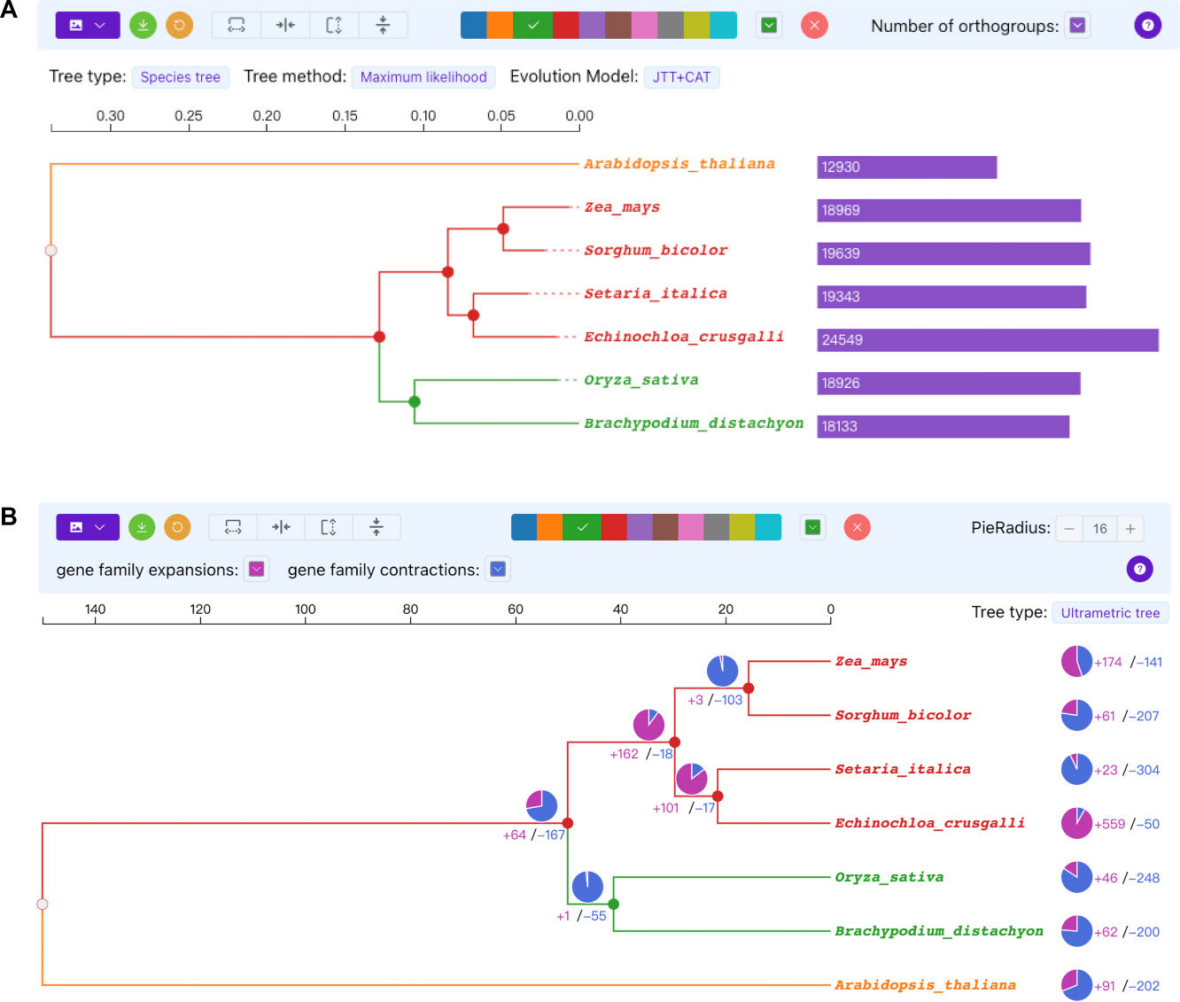
(**A**) A phylogenetic tree based on single-copy genes illustrates the evolutionary relationships and distances among the species. (**B**) A pie chart shows the number of gene families that have expanded (purple) or contracted (blue) during evolution, while the phylogenetic tree shows the evolutionary timeline of the species.

Simultaneously, OrthoVenn3 implemented a tree dendrogram feature that allows users to visualize phylogenetic trees with customizable styles that classify and label various developmental branches or nodes. Users can change the color of nodes and branches by clicking on the color block at the top of the diagram (see Figure 2A). The BarColor button can change the color of the statistical bar chart that displays the number of orthologous clusters for each species. Additionally, OrthoVenn3 supports specifying a species as the root node, which allows users to adjust the structural order of the phylogenetic tree. Users can export results in SVG and PNG formats with custom styles.

### Gene family contraction and expansion analysis function

Gene families are genes with similar structures and functions that can undergo expansions or contractions during evolution, potentially contributing to species differences (20,21). As such, analyzing gene family expansions and contractions is an essential part of genomic research (22). OrthoVenn3 now includes this functionality, offering a rich and customizable visualization approach that is both intuitive and interactive, with a wide range of options for presenting results.

OrthoVenn3 provides visualization for users to easily view the changes in the contraction and expansion of gene families. The gene family size can be visualized using a pie chart that shows the number of contracted (purple) and expanded (blue) gene families, intuitively displaying the evolutionary history of gene families and differences between species (Figure 2B). Users can customize the color and size of the pie chart by clicking the button To further understand the genetic mechanism behind the phenotypic differences of species, OrthoVenn3 supports GO term annotation of gene families with contractions and expansions. Users can view the functional annotation information by clicking on the numbers of the contracted or expanded gene families. Through gene family contraction and expansion analysis, users can gain insights into the evolutionary relationships between different species and the evolutionary history of gene families.

### Collinearity analysis function

**Figure 3.**
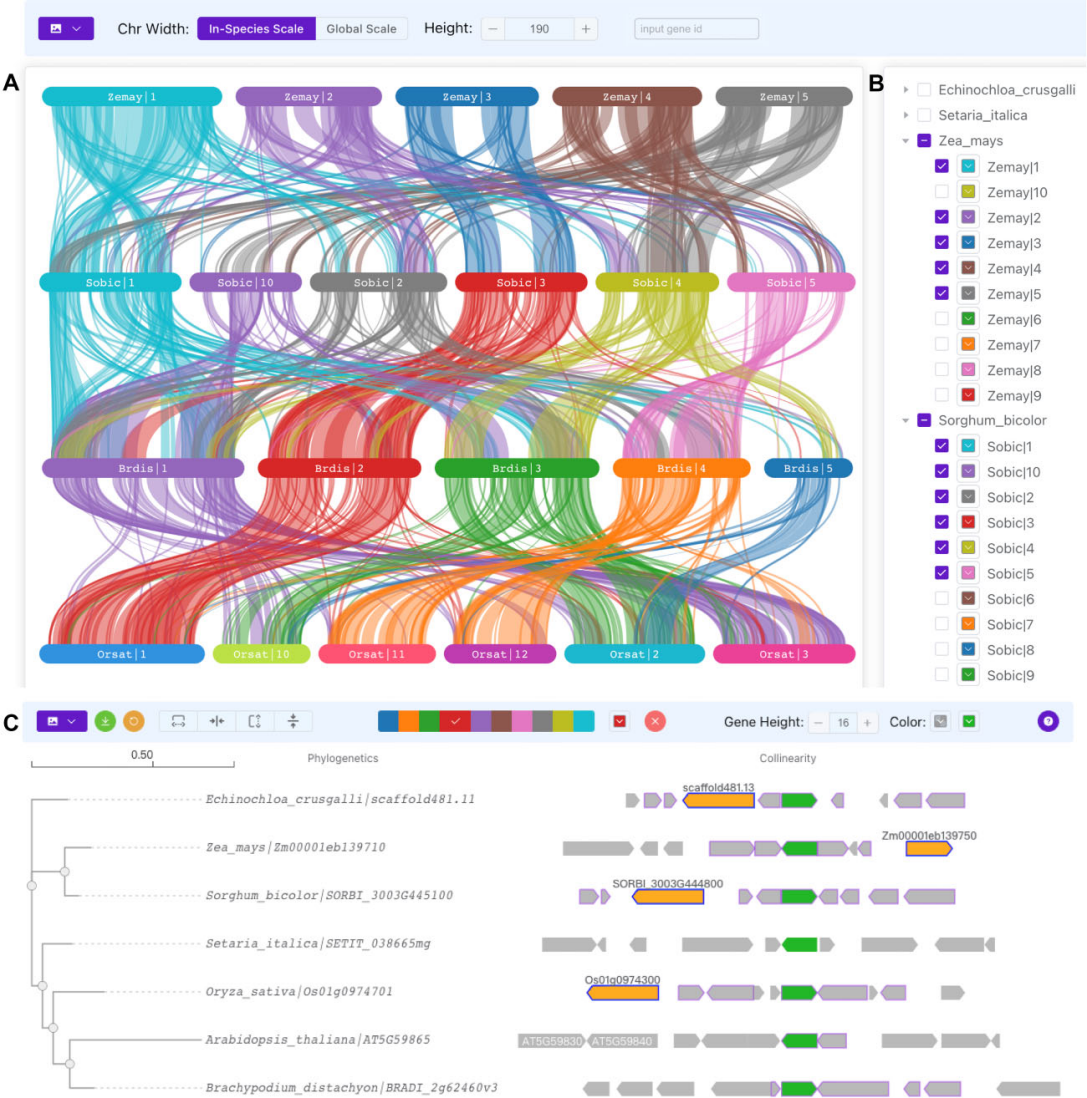
(**A**) A comparison of collinearity between adjacent species supports the highlighting of positions of genes of interest on the chromosome through the search box. (**B**) A list of chromosomes for each species allows the color of the chromosome to be changed by clicking on the color blocks. (**C**) Protein phylogenetic trees and collinearity within cluster15597 are shown. When the mouse hovers over a gene, the genes that are collinear with it are highlighted in orange.

Analyses of collinearity are crucial in evolutionary studies because they enable the detection and comparison of changes in genome structure and composition, including gene family expansions and transposon insertions (23). In our research, we utilized the MCScanX program (24) to identify collinearity between chromosomes of different species. This new feature enables us to compare the structure and composition of chromosomes across various species, which aids in understanding their evolutionary relationships and inferring chromosome rearrangements and evolution (25). Additionally, OrthoVenn3 provides two scaling models, the global scale and the in-species scale, to display collinearity. The global scale is suitable for displaying the collinearity of similar chromosome lengths based on the proportion of chromosome length among species, whereas the in-species scale focuses on the proportion of chromosome length within a single species. The in-species scale helps avoid incongruity of visualization effects caused by differences in chromosome length among different species, leading to improved quality of visualization results (Figure 3A, B). OrthoVenn3 allows users to search and label multiple genes on chromosomes simultaneously, enabling users to view the collinearity relationships among genes and their distribution regions on chromosomes.

OrthoVenn3 not only supports collinearity analysis for interspecies comparisons, but also for orthologous clusters. This feature enables users to identify the gain and loss of genes within orthologous clusters and investigate the functional evolution of gene families (26). The collinearity analysis results are presented with orthologous genes shown in green by default, while collinear genes are highlighted in orange by hovering over them (Figure 3C).

## CASE STUDY

To demonstrate the capabilities of OrthoVenn3, we conducted a comparative genomics study on *Echinochloa crus-galli*, focusing on its phylogenetic relationships and gene families annotated with environmental adaptation and invasion based on a recent study (27). Genomics data for *E. crus-galli* and six other plants were obtained from the Ensembl (2022 version) (10) database, and we employed the OrthoMCL algorithm for analysis. We used Diamond (28) with an *e* value of 1e-2 and set the split time of *A. thaliana–Z. mays* to 150 million years ago, and *O. sativa–Z. mays* to 50 million years ago. The results can be found at https://orthovenn3.bioinfotoolkits.net/result/b2f35873861c470f9f299b415e585044/orthologous.

The results revealed a total of 36 907 gene families, comprising 9052 highly conserved orthologous clusters and 28 265 families identified in the *E. crus-galli* genome. Notably, some gene families are involved in critical biological processes, such as signal transduction and growth regulation. The phylogenetic tree constructed from the analysis indicated that *E. crus-galli* is most closely related to *S. italica*, with an estimated split time of approximately 21.44 million years ago (Figure 2A, B).

Through the analysis of phylogenetic relationships, we gain insight into the temporal and spatial relationships of *Echinochloa crus-galli* during evolution, and changes in gene families may lead to changes in function. In our investigation of environmental adaptation and invasion, we identified 559 expanded gene families and 50 contracted gene families. These gene families were further annotated with GO terms and were found to be associated with functions such as monooxygenase activity, transferase activity, and glutathione metabolism. Previous studies (29) have demonstrated that these gene families are commonly associated with detoxification and weed resistance to synthetic herbicides, which supports the results of OrthoVenn3 annotation. Furthermore, OrthoVenn3’s collinearity analysis revealed that certain chromosomal regions of *E. crus-galli* have good collinearity with *Z. mays*, indicating that it may be their ancestral chromosomal regions. Other regions may have undergone rearrangement during evolution, providing insight into the evolutionary relationship and history between species.

## FUTURE DIRECTIONS

OrthoVenn3 is a versatile web-based tool for comparative genomics analysis that enables the analysis and visualization of genomics data in a single platform. However, traditional methods based on sequence similarity and topological structure may no longer meet the needs of orthologous cluster research (11,30). To overcome these limitations, the integration of OrthoVenn with deep learning technology is a promising direction for future research.

Deep learning is a machine learning method based on artificial neural networks that can automatically extract high-level features from data and achieve precise predictions through feature learning and model training (31). For the analysis of orthologous clusters, deep learning technology will be trained on sequence and structural features to achieve more accurate prediction and recognition of orthologous clusters. As deep learning technology can handle large-scale, complex, and high-dimensional data and perform transfer learning on different datasets, it has the potential to enhance the accuracy and efficiency of orthologous cluster identification and analysis.

Therefore, future versions of OrthoVenn are expected to leverage deep learning technology to become essential tools for comparative genomics research. By automatically extracting features from data, deep learning can provide more accurate predictions and enhance the analysis efficiency of orthologous clusters. This approach has great potential to improve our understanding of the evolution and function of genes across different species, leading to breakthroughs in biomedical research and biotechnology.

## DATA AVAILABILITY

The tool is freely accessible at https://orthovenn3.bioinfotoolkits.net.
